# Characterization and selection of Japanese electronic health record databases used as data sources for non-interventional observational studies

**DOI:** 10.1186/s12911-021-01526-6

**Published:** 2021-05-22

**Authors:** Yumi Wakabayashi, Masamitsu Eitoku, Narufumi Suganuma

**Affiliations:** 1 Integrated Center for Advanced Medical Technologies, Kochi Medical School, Kochi University, Kohasu, Oko-cho, Nankoku, Kochi, 783-8505 Japan; 2 Department of Environmental Medicine, Kochi Medical School, Kochi University, Kochi, Japan

**Keywords:** Real world, Retrospective study, Prospective study, Observational study, Virtual trial, Database, Medical information

## Abstract

**Background:**

Interventional studies are the fundamental method for obtaining answers to clinical questions. However, these studies are sometimes difficult to conduct because of insufficient financial or human resources or the rarity of the disease in question. One means of addressing these issues is to conduct a non-interventional observational study using electronic health record (EHR) databases as the data source, although how best to evaluate the suitability of an EHR database when planning a study remains to be clarified. The aim of the present study is to identify and characterize the data sources that have been used for conducting non-interventional observational studies in Japan and propose a flow diagram to help researchers determine the most appropriate EHR database for their study goals.

**Methods:**

We compiled a list of published articles reporting observational studies conducted in Japan by searching PubMed for relevant articles published in the last 3 years and by searching database providers’ publication lists related to studies using their databases. For each article, we reviewed the abstract and/or full text to obtain information about data source, target disease or therapeutic area, number of patients, and study design (prospective or retrospective). We then characterized the identified EHR databases.

**Results:**

In Japan, non-interventional observational studies have been mostly conducted using data stored locally at individual medical institutions (663/1511) or collected from several collaborating medical institutions (315/1511). Whereas the studies conducted with large-scale integrated databases (330/1511) were mostly retrospective (73.6%), 27.5% of the single-center studies, 47.6% of the multi-center studies, and 73.7% of the post-marketing surveillance studies, identified in the present study, were conducted prospectively. We used our findings to develop an assessment flow diagram to assist researchers in evaluating and choosing the most suitable EHR database for their study goals.

**Conclusions:**

Our analysis revealed that the non-interventional observational studies were conducted using data stored local at individual medical institutions or collected from collaborating medical institutions in Japan. Disease registries, disease databases, and large-scale databases would enable researchers to conduct studies with large sample sizes to provide robust data from which strong inferences could be drawn.

**Supplementary Information:**

The online version contains supplementary material available at 10.1186/s12911-021-01526-6.

## Background

During the course of primary-care medical practice, a huge amount of patient data, including laboratory results and diagnoses, administrative data, and health insurance information, are generated and collated as electronic health records (EHRs). Patients’ individual EHRs are then archived in databases that can be accessed by stakeholders throughout the medical field. Because these data arise through actual medical activities, they are considered real-world data; that is, these are observational data obtained through real-world medical practice rather than data obtained in an experimental setting.

**Fig. 1:**
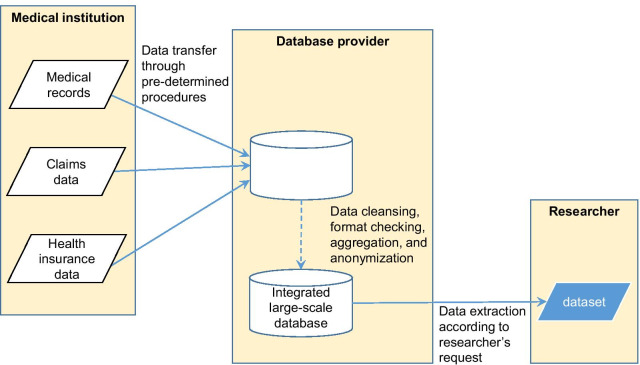
Overview of the construction of a large-scale, integrated, electronic health record (EHR) database. White parallelograms represent the data included in EHRs. Cylinders represent EHR databases. The dark parallelogram represents the dataset provided to researchers who conduct non-interventional observational studies

Traditionally, interventional studies are the fundamental method for obtaining answers to clinical questions. In such studies, researchers enroll patients, randomize them into two or more groups, provide the groups different medical treatments, and compare the resulting data between groups. However, it is sometimes difficult to conduct interventional studies because of insufficient financial or human resources or the rarity of the disease in question. One means of addressing these issues is to conduct a non-interventional observational study using EHR databases as the data source. Such data sources can range from EHRs obtained from individual medical institutions to large-scale integrated EHR databases organized and maintained by database providers (Fig. 1). Large-scale databases allow researchers to conduct non-interventional observational studies with a large sample size, thus affording robust real-world evidence [1, 2]. Using large-scale databases is also a quick way to analyze the real-world clinical situation at a modest cost [3].

Some large-scale integrated EHR databases in Japan include claims data as well as medical records [4–6]. Claims data provides information about diagnoses, prescriptions, medical care, and costs.

When planning a non-interventional observational study using a EHR database, researchers must first identify the databases that are most suitable for their study purpose. Although many such databases are available [4, 5], they each have their strengths and limitations that must be considered and weighed against one another. For example, most EHRs, and therefore most databases, lack patient data regarding the pre-symptomatic stage of disease; therefore, many EHR databases will be of limited benefit to researchers who intend to investigate aspects of the early stages of disease onset such as the pre-symptomatic stage of Alzheimer’s disease. This lack of data likely reflects that, despite the potential benefits of early preventive care [7–10], patients in the pre-symptomatic stage of disease are rarely seen in hospital. Researchers also need to consider important database characteristics such as the number of records, accessibility to outcome data, duration of follow-up, and potential biases during data collection [11]. How best to evaluate the suitability of an EHR database when planning a study remains to be clarified.

The aim of the present study was to identify and characterize the data sources that have been used for conducting non-interventional observational studies in Japan and to propose a flow diagram to help researchers assess the suitability of a potential EHR database for use in their non-interventional observational study.

## Methods

### Determination of the data sources used for non-interventional observational studies in Japan

We assessed published articles reporting non-interventional observational studies conducted in Japan in order to determine the data sources for the studies. We used a two-part approach to identify articles: the first part involved searching PubMed; the second part involved collecting articles from the websites of large-scale database providers. We referred to, and followed, the PRISMA (Preferred Reporting Items for Systematic reviews and Meta-Analysis) Statement [12] to identify and screen articles, specifically the Identification and Screening phases of the PRISMA Flow. Then we selected and classified articles through our own methods because the remaining components of the PRISMA Statement were not fully applicable to our review targeting non-observational studies.

In the first part of our study, we conducted a PubMed search to find articles reporting observational studies conducted in Japan in the last 3 years; the following settings were used: “Japan” was set as the keyword in the Affiliation field, “observational study” was set in the filters for article type, “from December 1, 2017, to November 30, 2020” was set for publication date, “humans” was set for species, and “English” was set for language.

Once candidate articles were identified, we reviewed their abstracts. We excluded articles without an abstract or without a structured abstract. Structured abstracts provide more informative summaries than non-structured abstracts [13].

We reviewed each abstract for information about data source, target disease or therapeutic area, number of patients enrolled in the study, and study design. If the abstract did not include this information, we reviewed the full text of the article. If the full text was not available or lacked enough information, we excluded the article. We also excluded:

- articles reporting studies conducted multi-nationally or in a country other than Japan. We consider that medical practices and the information contained within EHRs could differ between regions.
- articles reporting non-clinical studies, interventional studies, or studies with healthy subjects or controls.

Classification of each article was conducted using the following criteria:

*Data source*: Articles were classified into four types based on the source data used: (1) data stored at a medical institution, (2) data collected from several medical institutions, (3) data obtained from a disease registry or database, (4) data obtained from a large-scale integrated database.

1. When the words “single-center study” were included in the title, abstract, or full text, the article was classified as type (1), as were articles that similarly contained the phrase “single center”, “single-center”, “single centre”, “single-centre”, “single institution”, or “single-institution”. When an article included the words indicating single-center study such as “in a xxxx [adjective] hospital”, “in our hospital”, “at our hospital”, “in our institution”, “at our institution”, “at our institute”, “at XXXX [institution name]”, “in XXXX [institution name]”, or “in a xxxx [adjective] center”, the article was classified as type (1).
2. When the abstract or full text included the words “multi-center study” or “xx [digit]-center study”, the article was classified as type (2). Because post-marketing surveillance (PMS) studies are a kind of multi-center study, these articles should also be classified as (2). However, in Japan, PMS studies are conducted under Good Post-marketing Study Practice (GPSP) regulations [14], whereas observational studies are conducted under the ethical guidelines of the Japan Ministry of Education, Culture, Sports, Science and Technology [15]. Thus, we considered PMS studies separately from the other multi-center studies.
3. When the abstract or full text included “registry” or “study database”, the article was classified as type (3). Articles reporting a multi-center study to construct and/or leverage a disease registry or database was classified as (3).
4. When the abstract or full text included “claims database”, the article was classified as type (4). We classified articles with phrases such as “nationwide database” as type (3) or (4) depending on the name of the database that actually was used, if it was available elsewhere in the abstract or full text. An article reporting several types of studies including single-center or multi-center studies as well as large-scale database analyses, were classified as (4).

*Target disease or therapeutic area*: Articles were classified according to the following 19 classes; these classes reflect our own classification criteria, which we developed by using the 10th revision of the International Statistical Classification of Diseases and Related Health Problems (ICD-10) [16]: (1) infectious and parasitic diseases other than coronavirus disease 2019 (COVID-19), (2) COVID-19, (3) cancer and neoplasm, (4) diseases of the blood and blood forming organs, (5) endocrine, nutritional and metabolic diseases, (6) diabetes, (7) mental disorder, (8) disease of the nervous system, (9) disease of the eye and adnexa, (10) disease of the ear and mastoid process, (11) disease of the circulatory system, (12) disease of the respiratory system, (13) disease of the digestive system, (14) Disease of the skin and subcutaneous tissue, (15) disease of the musculoskeletal system and connective tissue, (16) disease of the genitourinary system, (17) pregnancy, childbirth, and perinatal, (18) injury or other consequences of eternal causes, (19) others, including surgery, transplantation, hemodialysis, dental, and pain.

By referring to ICD-10, we classified cardiac surgery as “injury, poisoning, and certain other consequences of external causes” and not as “disease of the circulatory system”. We applied this approach to the study focuses as well. For example, an article reporting retinal disorders in diabetic patients was classified as (9) disease of the eye and adnexa and not as (6) diabetes.

*Number of patients enrolled in study:* The number of patients included in the final analysis was obtained by reviewing the abstract or full article text.

*Study design*: Studies were classified as either prospective or retrospective, depending on which word was used in the abstract or full article text. We classified articles as “prospective” or “retrospective” according to the article author’s definition. Thus, we classified an article as “unknown” without making a conjecture when it did not mention either “prospective” or “retrospective”.

The review author identified the articles, extracted information from the articles, and summarized the information into an extraction sheet so that the article could be classified. Another author checked the appropriateness of the classification methodology. The review author then self-checked the classification results regarding the classification of the data source and study design. The information about the data source was found in the article title (16%), abstract (40%), or main body (44%).

In the second part of our study, we obtained a list of articles reporting observational studies using data from one or more of four large-scale Japanese EHR databases [17–21]: Japan Medical Data Center Claims Database (JMDC Claim), Medical Data Vision Database (MDV Database), National Database of Health Insurance Claims and Specific Health Checkups of Japan (NDB Japan), and Medical Information Database Network (MID-NET). JMDC Claim and MDV Database are the largest EHR databases in Japan [4]. NDB Japan and MID-NET are widely known databases in Japan that are provided by the Japanese governmental organization. These four databases all include EHR data generated by the Japanese Diagnosis Procedure Combination/Per-Diem Payment System (DPC; the Japanese medical payment framework); therefore, these databases are sometimes colloquially referred to as “DPC databases”. For example, NDB Japan is frequently called “the DPC database” because it is the most well-known database in Japan.

To obtain this information, we accessed the list of published articles available at the website associated with each of the four databases. For JMDC Claim and MDV Database, the publication lists were very long, so we limited our search to the period January 2017 through December 2020. For NDB Japan and MID-NET, the publication lists only included articles published since 2018.

We reviewed each abstract for information about the target disease or therapeutic area, number of patients enrolled in the study, and study design, by using the same classification criteria described above. Because these publication lists were from large-scale databases, data source classification was not needed. If the abstract of an article did not include information regarding the target disease or therapeutic area, number of patients enrolled in the study, and study design, we reviewed the full text of the article. When the full text was unavailable or lacked sufficient information, we excluded the article. We also excluded those articles that overlapped with the articles selected through the PubMed search or another database provider’s site. Finally, we calculated the number of articles classified and compiled the results of the PubMed search and database website investigation.

### Characteristics of the large-scale integrated databases

By contacting the relevant organizations through their websites, we solicited information regarding the procedures through which the four large-scale databases were established. JMDC Claim was constructed by the Japan Medical Data Center Inc. and comprises EHR data collected through daily medical practice since 2005. The MDV Database and related services have been maintained by Medical Data Vision Co., Ltd., since 2008. NDB Japan was constructed by the Japan Ministry of Health, Labour and Welfare (MHLW) in 2009. MID-NET has been offered through a governmental organization (under the MHLW) and the Pharmaceuticals and Medical Devices Agency since 2018. In characterizing these databases, we focused on the (1) data sources and procedures for processing data, (2) data items available, and (3) anonymization of data. We also contacted the companies responsible for two of the databases (Japan Medical Data Center and Medical Data Vision) to ask for general information regarding the construction and maintenance of their databases; the same questions regarding data cleaning, data standardization, and database construction were sent to both companies via e-mail messages, not by using any questionnaire form.

### Development of a flow diagram for evaluating and choosing databases

We summarized our findings regarding EHR database characteristics and published literature based on studies conducted by using the databases. By considering the available data items, reliability, anonymization, data volume, and subject follow-up period of these datasets, we developed an assessment flow diagram as a tool for evaluating databases and choosing those suitable for the intended use.

## Results

### Determination of the data sources used for non-interventional observational studies in Japan

**Fig. 2:**
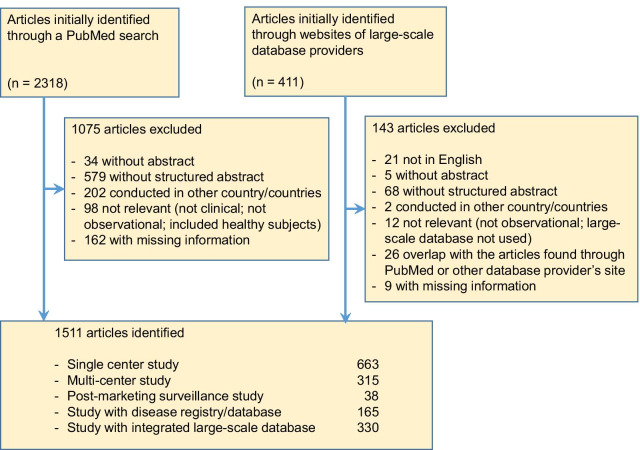
Results of our screening of observational studies in Japan

Of 2729 articles identified (2318 through PubMed and 411 through the websites of the large-scale database providers), 1511 articles met the eligibility criteria (Fig. 2). The data sources used in the identified studies are shown in Table 1, stratified by study type.

**Table 1:** Summary of the data source used for non-interventional observational studies conducted in Japan

Study type	Data source	Database example	Strengths	Limitations
Single-center study	Data stored at a single medical institution	-	Medical practice well-known Flexible approach	Limited volume of data [28]
Multi-center study	Medical records collected from collaborating medical institutions	-	Study team collaboration	Selection bias [29, 30] Medical practices can differ by institution [30]
Post-marketing surveillance study^a^	Medical records collected from participating medical institutions	-	Data volume	Cost [31]
Study using a disease registry/database	Disease registry Disease database	All Japan Utstein Registry [22, 23] NinJa [24, 25] KCHF Registry [26, 27]	Disease-specific test results available Data volume	Selection bias [27]
Study using a dataset from a large-scale integrated database	Integrated large-scale database	JMDC Claim^b^ MDV Database^c^ NDB Japan^d^ MID-NET^e^	Data volume	Careful interpretation of data is needed [31]

*PMS* Post-marketing surveillance, *GPSP* Good Post-marketing Study Practice, *NinJa* National Database of Rheumatic Diseases by iR-net (Divison of Rheumatology, Immunologic Disorder Network, National Hospital Organization) in Japan, *KCHF* *Registry* Kyoto Congestive Heart Failure Registry, *JMDC* Japan Medical Data Center, *MDV* Medical Data Vision, *NDB Japan* National Database of Health Insurance Claims and Specific Health Checkups of Japan, *MID-NET* Medical Information Database Network

^a^A PMS study is a kind of multi-center study initiated by a sponsor and conducted under GPSP regulations [14, 15]

^b^JMDC Claim is provided by Japan Medical Data Center, Inc. [17]

^c^MDV Database is provided by Medical Data Vision Co., Ltd. [18]

^d^NDB Japan is provided by the Ministry of Health, Labour and Welfare of Japan [19, 20]

^e^MID-NET is sponsored by the Pharmaceuticals and Medical Devices Agency of Japan [21]

A total of 663 (43.9%) studies were single-center studies. In these studies, the sample size was < 100 in 278 (41.9%) studies and 100–299 in 214 (32.3%) studies (Table 2, Additional files 1 and 2). Thus, 74.2% of the single-center studies used a sample size < 300. The single-center studies were conducted in various target diseases and therapeutic areas.

**Table 2:** Characteristics of the non-interventional observational studies identified in the present study

Characteristic	Single-center study		Multi-center study		PMS study		Study using a disease registry/database		Study using a large-scale integrated database	
Subtotal (percentage of total article number [1511])	663	(43.9)	315	(20.8)	38	(2.5)	165	(10.9)	330	(21.8)
Study design	n	%	n	%	n	%	n	%	n	%
Prospective	182	27.5	150	47.6	28	73.7	62	37.6	6	1.8
Retrospective	366	55.2	110	34.9	0	0	68	41.2	243	73.6
Unknown^a^	115	17.3	55	17.5	10	26.3	35	21.2	81	24.5
Target disease or therapeutic area										
Infectious and parasitic diseases other than COVID-19	31	4.7	18	5.7	0	0	7	4.2	34	10.3
COVID-19	2	0.3	0	0	0	0	0	0	0	0
Cancer and neoplasm	74	11.2	56	17.8	6	15.8	16	9.7	28	8.5
Diseases of the blood and blood forming organs	2	0.3	5	1.6	2	5.3	0	0	1	0.3
Endocrine, nutritional and metabolic diseases	9	1.4	1	0.3	0	0	2	1.2	12	3.6
Diabetes	20	3.0	10	3.2	6	15.8	3	1.8	48	14.5
Mental disorder	6	0.9	5	1.6	0	0	3	1.8	11	3.3
Disease of the Nervous system	21	3.2	8	2.5	1	2.6	2	1.2	14	4.2
Disease of the eye and adnexa	43	6.5	10	3.2	1	2.6	0	0	2	0.6
Disease of the ear and mastoid process	3	0.5	1	0.3	1	2.6	0	0	0	0
Disease of the Circulatory system	124	18.7	49	15.6	7	18.4	79	47.9	41	12.4
Disease of the Respiratory system	32	4.8	11	3.5	1	2.6	7	4.2	12	3.6
Disease of the Digestive system	25	3.8	18	5.7	1	2.6	0	0	15	4.5
Disease of the skin and subcutaneous tissue	6	0.9	1	0.3	0	0	2	1.2	4	1.2
Disease of the musculoskeletal system and connective tissue	18	2.7	20	6.3	8	21.1	13	7.9	15	4.5
Disease of the genitourinary system	24	3.6	16	5.1	2	5.3	3	1.8	9	2.7
Pregnancy, childbirth, perinatal	19	2.9	8	2.5	0	0	3	1.8	9	2.7
Injury, other consequences of eternal causes	34	5.1	18	5.7	0	0	10	6.1	14	4.2
Others	170	25.6	60	19.0	2	5.3	15	9.1	61	18.5
Patient number										
99≦	278	41.9	70	22.2	1	2.6	4	2.4	2	0.6
100–299	214	32.3	83	26.3	4	10.5	19	11.5	9	2.7
300–999	118	17.8	83	26.3	9	23.7	42	25.5	27	8.2
1000–2999	36	5.4	53	16.8	9	23.7	28	17.0	40	12.1
3000–9999	10	1.5	20	6.3	13	34.2	29	17.6	59	17.9
10,000–99,999	6	0.9	5	1.6	2	5.3	23	13.9	95	28.8
≧100,000	0	0	1	0.3	0	0	20	12.1	98	29.7

*COVID-19* coronavirus disease 2019, *PMS* post-marketing surveillance

^a^Words to indicate study design classification, prospective or retrospective, were not found in the article

A total of 315 (20.8%) studies were multi-center studies. In these studies, the sample size was < 100 in 70 (22.2%) studies, 100–299 in 83 (26.3%) studies, and 300–999 in 83 (26.3%) studies. Thus, 74.8% of the multi-center studies used a sample size < 1000. The multi-center studies were also conducted in various target diseases and therapeutic areas.

A total of 38 PMS studies were identified. Generally, PMS studies are used to gather information about a new medicinal product or medical device after it has been granted marketing authorization; thus, 28 of the 38 PMS studies (73.7%) were conducted prospectively. In the 39 PMS studies, the sample size was 1000–2999 in 9 (23.7%) studies, 3000–9999 in 13 (34.2%) studies, and ≥ 10,000 in 2 (5.3%) studies.

A total of 165 studies conducted for the construction of a disease registry or database were identified. Such studies are usually conducted prospectively, although the disease registries or databases themselves are sometimes used for retrospective secondary analysis. In the identified studies, three Japanese registries/databases were used by researchers of different studies: All-Japan Utstein Registry [22, 23], National Database of Rheumatic Diseases by iR-net (Division of Rheumatology, Immunologic Disorder Network, National Hospital Organization) in Japan (NinJa) [24, 25], and Kyoto Congestive Heart Failure (KCHF) Registry [26, 27]. In addition, although our focus was on studies conducted in Japan, we identified seven registry studies conducted by using the Surveillance, Epidemiology, and End Results (SEER) program of the US National Cancer Institute.

Finally, we identified 330 studies using large-scale integrated databases, 243 (73.6%) of which were conducted retrospectively. The sample size was 10,000–99,999 in 95 (28.8%) studies and ≥ 100,000 in 98 (29.7%) studies. In the identified studies, the following large-scale integrated databases were used: JMDC Claim, 152 studies; MDV Database, 130 studies (9 were studies using both JMDC Claim and MDV Database); NDB Japan, 17 studies; MID-NET, 2 studies. In the remaining articles, the databases used were referred to only as “DPC database” and the actual names of the databases were not mentioned. The identified data sources are shown as examples in Table 1. Each data source has its strengths and limitations [27–31].

### Characteristics of large-scale integrated databases

Summaries of the four large-scale integrated databases are shown in Table 3. NDB Japan is one of several Japanese DPC databases. MID-NET was launched 2018 and includes laboratory data as well as DPC information. The data sources, available data items, and anonymization status were obtained from the websites of the respective databases [17–21].

**Table 3:** Japanese large-scale integrated databases and their characteristics

	JMDC Claim^a^	MDV Database^b^	NDB Japan^c^	MID-NET^d^	Points to consider when selecting a large-scale database
Characteristics of the database	Information regarding health insurance claims and DPC collected from institutions through standardized procedures since 2005	Claims information, DPC information, and laboratory data collected from institutions through standardized procedures since 2008	Claim information on medical treatment, dental treatment, medications, and DPC Constructed in 2009 Data of subjects over 65-year-olds are available Dataset service has been offered since 2011	Medical records, health insurance claims, and DPC information Data from 23 medical sites collected and verified Dataset service has been offered since 2018	The database should be established through data-processing procedures that are satisfactorily explained and reproducible. Data for intended follow-up period is important
Data volume	5.6 million subjects (as of June 2018)	2.8 million subjects (as of May 2019)	All Japanese citizens (not clearly described)	4 million subjects (as of November 2018)	Having sufficient data to support an appropriate sample size is crucial
Available data	Information regarding health insurance, claims, DPC; medical products’ information coding; diagnosis; number of patients; complications; surgery; medication information; and events Data from healthy subjects are available Data are updated monthly according to pre-defined processes	Information on claims and DPC and laboratory data are available Data updated monthly according to pre-defined processes	Claim information on medical treatment, dental treatment, medications, and DPC Use of NDB Japan is permitted for evaluated researchers only NDB Open Data Japan, partial dataset, is freely available	Demographic data; hospital visits/admissions; disease and injury diagnoses; medical treatment, laboratory data, results of physiologic tests; pharmacologic data; and medications	Information related to patients and exposures, such as diagnosis, medications, and surgery, should be available
Number of published articles	304 articles published (as of December 2020)	213 articles published (as of December 2020)	78 articles published^e^ (as of October 2020)	11 articles published (as of April 2020)	
Anonymization?	Anonymized	Anonymized	Subjects’ personal information is not available. Anonymization is up to the individual researcher	Anonymized	Appropriate anonymization of data is important to adhere to ethics and quality standards

*JMDC* Japan Medical Data Center, *MDV* Medical Data Vision, *NDB Japan* National Database of Health Insurance Claims and Specific Health Checkups of Japan, *MID-NET* Medical Information Database Network, *DPC* Diagnosis Procedure Combination/Per-Diem Payment System (Japanese medical payment framework)

^a^JMDC Claim is provided by Japan Medical Data Center, Inc. [17]

^b^MDV Database is provided by Medical Data Vision Co., Ltd. [18]

^c^NDB Japan is provided by the Ministry of Health, Labour and Welfare of Japan [19], which also provides NDB Open Data Japan [20]

^d^MID-NET is sponsored by the Pharmaceuticals and Medical Devices Agency of Japan [21]

^e^The article list of NDB Japan shows 119 items including technical reports and congress abstracts for oral or poster sessions as well as published articles

Health insurance companies transfer claims data derived at medical institutions to JMDC Claim according to pre-defined procedures once a month. Similarly, MDV receives anonymized data from medical institutions on a monthly basis; the data managers then check the data and update the database. According to the MID-NET website, medical records and claims data are transmitted to the Integrated Data Source of MID-NET through pre-defined procedures. The MID-NET system and data are monitored and verified at variable intervals.

The detailed data processing procedures for NDB Japan are not shown on the information website offered by the Japan Ministry of Health, Labour, and Welfare (MHLW) [19]. Although, the website states that the use of the NDB Japan data is usually permitted for academic researchers only, MHLW does extract part of the NDB Japan data to create a small dataset called NDB Open Data Japan, which is freely available at the MHLW website [20].

### Development of a flow diagram for evaluating and choosing databases

**Fig. 3:**
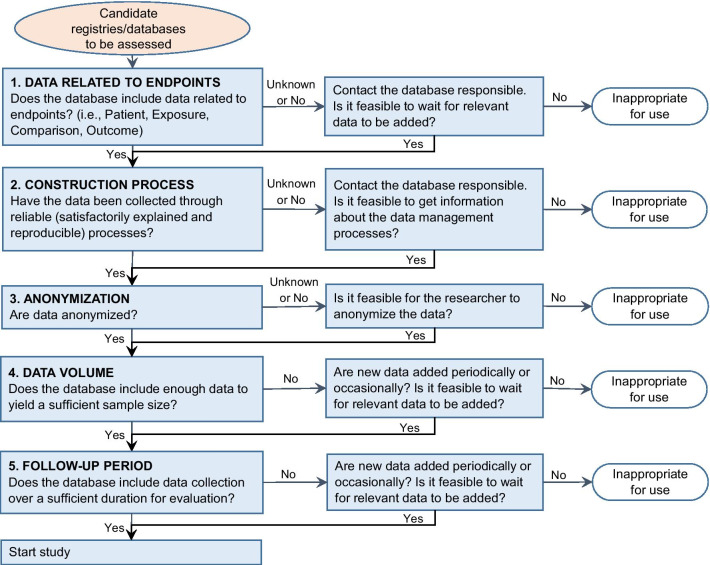
Flow diagram for the assessment of the suitability of a registry or database for use in a non-interventional observational study

Using our combined findings, we developed an assessment flow diagram to identify EHR databases appropriate for various applications (Fig. 3). The order of steps was determined according to the consistency with the researcher’s study purpose, and the quality and quantity of the data.

1. DATA RELATED TO ENDPOINTS: According to our interpretation of the publications’ data, the most important characteristic of an EHR database in terms of its suitability for a non-interventional observational study is that parameters related to endpoints are available. For example, Horii et al. used the MDV Database to reveal that patients with type 2 diabetes who were treated with sodium–glucose cotransporter 2 inhibitors had an increased risk of developing hypoglycemia [32]. The authors were able to perform their study [32] because the MDV database includes information about diagnosis, medications, and patient background such as body mass index and glycohemoglobin (HbA1c). In addition, Takeuchi et al. identified the relationship between hemoglobin and HbA1c by analyzing the JMDC Claim database [33]. Momo et al. also used the JMDC Claim database and reported that compliance with statin therapy needs to be improved among working-age male patients treated with statin and anti-platelet drugs, especially in those patients with increased baseline low-density lipoprotein C [34]. Finally, Koretsune et al. used the MDV Database to investigate cardiovascular event occurrence in patients treated with dabigatran (oral anticoagulant) or warfarin [35]. In all of these example studies, relevant parameter data were available in the databases. To be suitable for a researcher’s non-interventional study, the selected database should contain patient data, including demographic information and details regarding medication [32, 35], surgery [36], and hospitalization [32, 35].
2. CONSTRUCTION PROCESS: For use in non-interventional studies, the database should be established through data-processing procedures those are satisfactorily explained and reproducible. The database construction processes of JMDC Claim, MDV Database, and MID-NET are explained on their websites [17–21].
3. ANONYMIZATION: Appropriate anonymization of data is important from ethics and quality standpoints. Regional regulations regarding anonymization must be accommodated. Data anonymization of JMDC Claim, MDV Database, and MID-NET are mentioned on their websites. Subjects’ personal information is not available for researchers using NDB Japan, and their website states that anonymization is the researcher’s responsibility [19, 20].
4. DATA VOLUME: Having sufficient data to support an appropriate sample size is crucial for robust non-interventional studies. Even when a potential database lacks a sufficient volume of data initially, a researcher might still consider the database when the data-processing processes are known and the volume of data is likely to increase. The data volumes currently available in the JMDC Claim, MDV Database, and MID-NET are summarized in Table 3; data updates are announced on their websites.
5. FOLLOW-UP PERIOD: Having sufficient data that span the researcher’s intended follow-up period is an important feature of a candidate database. Researchers sometimes need long-term information [37]. When data for the scheduled period are unavailable initially, researchers have the option of waiting for data updates to accumulate longer-term data. However, missing data might ultimately be unavoidable. For JMDC Claim, data has been collected since 2005, for MDV Database since 2008, and for NDB Japan since 2009.

### Examples of using the assessment flow diagram

First, consider a researcher who wants to conduct a study to answer the question “Does physicians’ prescription behavior change after they receive a ‘Dear Doctor’ letter?” After referring to the MID-NET website, the researcher is able to confirm the appropriateness of leveraging MID-NET as a potential EHR database for the study by answering ‘yes’ to Questions 1 through 5 of the assessment flow diagram (Fig. 3). If, while assessing JMDC Claim or MDV Database, the researcher needs more information than is available on their websites, the researcher can contact JMDC or MDV representatives.

In contrast, a researcher interested in evaluating patients’ quality of life will likely respond ‘no’ to Question 1 regarding large-scale integrated databases with primary-care information, such as JMDC Claim, MDV Database, MID-NET, and NDB Japan. Therefore, this investigator needs to find other registries that contain data appropriate for quality-of-life evaluation.

## Discussion

We investigated the data sources that have been used for conducting past non-interventional observational studies in Japan. We also characterized four Japanese large-scale integrated EHR databases by summarizing their data sources, available data, and anonymization status.

We found that non-interventional observational studies in Japan are mostly conducted by using data stored at individual medical institutions or collected from several collaborating medical institutions. This approach provides a limited volume of data. Using a disease registry, disease database, or large-scale integrated EHR database might be a way to increase the sample size. Such registries and databases have the advantage that the data are already anonymized and cleaned.

Observational studies using large-scale databases are usually conducted retrospectively. However, we found that 27.5% of the single-center studies, 47.6% of the multi-center studies, and 73.7% of the PMS studies, identified in the present study, were conducted prospectively. When a researcher focuses on a new parameter as a study endpoint, they must take a prospective approach because no data for the parameter is yet included in medical records at their medical institution or in integrated large-scale databases. However, if the target parameter data is expected to be collected and added to EHRs and EHR databases in the future, the researcher may wait for further data accumulation.

Applying our present findings, we developed a flow diagram that can be used to assess the suitability of a registry or database for use in a non-interventional observational study (Fig. 3). In this context, it is crucial that the EHR database contains data related to the study endpoints, such as laboratory findings and the treatments (exposures) the patients received. The database should also contain demographic information as well as patients’ medication, surgery, and hospitalization histories.

When planning a non-interventional observational study, it is helpful for researchers to know how a database is constructed and what anonymization processes have been applied to the data. Appropriate anonymization of data is important to adhere to current ethics and quality guidelines. Also, the database should have been established through data-processing procedures that are satisfactorily explained and reproducible. The database construction and data anonymization processes of JMDC Claim, MDV Database, and MID-NET are explained on their websites [17, 18, 21].

Having sufficient data to support an appropriate sample size is crucial to obtain robust outcomes. Even when a potential database lacks a sufficient volume of data initially, a researcher might still consider using the database when the data-processing approach is known and the volume of data is expected to increase. If the patient follow-up period is too short to extract relevant data to cover the duration of the intended study, researchers have the option of waiting for data updates to accumulate longer-term data. However, missing data might ultimately be unavoidable. For JMDC Claim database, data has been collected since 2005, for MDV Database since 2008, and for NDB Japan, since 2009.

PMS studies are performed by taking a prospective approach to gather information about a new medicinal product or medical device after it has been granted marketing authorization. The MHLW, a ministry of the Japanese Health Government, implemented the MID-NET system and intended that it would become a major data source for PMS studies [6, 38]. In addition, by comparing MDV dataset analysis with their prospective PMS study, Sakata et al. demonstrated that large-scale database analysis with MDV Database could be useful in long-term drug safety assessment; these authors also mentioned that using a database decreased the time needed to complete a PMS study and was relatively inexpensive [31]. Data related to exposure to a new medicinal product is very limited in EHRs at the time when planning a PMS study but is collected and added to EHRs at medical institutions and then collated into large-scale integrated databases over time. If a PMS researcher or sponsor can wait for the data to be accumulated, they can use a large-scale database to conduct their study with less financial or human resource costs. In fact, a researcher who intends to conduct a prospective study can use a large-scale database by employing the tactic of waiting for data accumulation of the database. Prospective clinical studies are rigidly managed, which usually means huge amounts of human and financial resources are needed [39, 40].

We acknowledge several limitations to the present study. First, we identified the articles reporting observational studies through PubMed searches and database providers’ publication lists, instead of accessing clinical trial registration sites directly. This strategy means we didn’t focus on all studies those have been planned and initiated in Japan, but our analysis was based on information about completed and published studies. Second, our group has not yet conducted an observational study using the EHR databases evaluated in the present study; in the next phase of our research, we intend to conduct a study using an appropriate EHR database. Finally, we do not address limitations regarding linking Japanese EHR data stored on different platforms, such as the various health insurance databases.

## Conclusion

Our analysis revealed that the non-interventional observational studies were mostly conducted using data stored local at individual medical institutions or collected from collaborating medical institutions in Japan. Disease registries, disease databases, and large-scale integrated EHR databases would enable researchers to conduct studies with large sample sizes to provide robust data from which strong inferences could be drawn. Using our flow diagram, researchers planning non-interventional observational studies should consider the strengths and limitations of each available database and choose the most appropriate one to their study goals. Whereas observational studies using large-scale databases are usually retrospective, a researcher, even planning a prospective study, can leverage a large-scale database by employing the tactic of waiting for data accumulation of the database.

## Supplementary Information

**Additional file 1**. pubmed_literature_observational_Japan_3_years_human_2020. The list of articles identified through PubMed search includes article title, authors, journal, data source, therapeutic area, patient number, and study design.

**Additional file 2**. MDV_JMDC_NDB_MIDNET_literature_2020. The list of articles identified through websites of large-scale database providers includes article title, authors, journal, therapeutic area, patient number, and study design.

## Data Availability

The datasets used and/or analyzed during the present study are available from the corresponding author of reasonable request. The identified articles information is available as Additional files 1 and 2.

## Abbreviations

COVID-19: Coronavirus disease 2019
DPC: Diagnosis Procedure Combination/Per-Diem Payment System
EHR: Electronic health record
GPSP: Good Post-marketing Study Practice
ICD-10: The 10th revision of the International Statistical Classification of Diseases and Related Health Problems
HbA1c: Glycohemoglobin
JMDC: Japan Medical Data Center
KCHF: Kyoto Congestive Heart Failure
MDV: Medical Data Vision
MHLW: Ministry of Health, Labour, and Welfare
MID-NET: Medical Information Database Network
NDB Japan: National Database of Health Insurance Claims and Specific Health Checkups of Japan
NinJa: National Database of Rheumatic Diseases by iR-net (Division of Rheumatology, Immunologic Disorder Network, National Hospital Organization) in Japan
PMS: Post-marketing surveillance
PRISMA: Preferred Reporting Items for Systematic reviews and Meta-Analyses
SEER: Surveillance, Epidemiology, and End Results program
